# A survey of influence of work environment on temporomandibular disorders-related symptoms in Japan

**DOI:** 10.1186/1746-160X-8-24

**Published:** 2012-09-21

**Authors:** Akira Nishiyama, Koji Kino, Masashi Sugisaki, Kaori Tsukagoshi

**Affiliations:** 1 Section of Temporomandibular Joint and Oral Function, Department of Comprehensive Patient Care, Graduate School, Tokyo Medical and Dental University, 1-5-45, Yushima, Bunkyo-ku, Tokyo, 113-8549, Japan; 2 Department of Dentistry, Jikei University School of Medicine, Tokyo, Japan

**Keywords:** Temporomandibular disorders, Work environment, Questionnaire, Personal computer use

## Abstract

**Introduction:**

This study aimed at identifying the factors that influence the incidence of temporomandibular disorders (TMD)-related symptoms (TRS) in a Japanese working population.

**Methods:**

Our study subjects comprised of 1,969 employees from the same Japanese company. The subjects were assessed using a questionnaire that covered both TRS and the work environment. TRS were measured from 4 items on the questionnaire. The work environment factors recorded were the daily mean duration of personal computer use, driving, precise work, commuting, time spent at home before going to bed, sleeping, attending business meetings, and performing physical labor. Statistical analysis was performed using *t*-tests, Chi-square tests, and logistic regression analyses. A result with *P* < 0.05 was considered statistically significant.

**Results:**

The median total score on the 4 items used to assess TRS was 5 (25% = 4, 75% = 7). Two groups were defined such that the participants scoring ≤7 were assigned to the low-TRS group and those scoring ≥8, to the high-TRS group. The high-TRS group constituted 22.6% of the subjects. Logistic regression analyses indicated that female gender and extended periods of computer use were significant contributors to the manifestation of TRS.

**Conclusion:**

This questionnaire-based study showed that gender and computer use time was associated with the prevalence of TRS in this working population. Thus, evaluation of ergonomics is suggested for TMD patients.

## Introduction

The term “temporomandibular disorders (TMD)” encompasses a number of clinical conditions that involve the temporomandibular joint, the masticatory muscles, or both [1]. The prevalence of TMD in the general population has been reported to be 5%–12% [2,3]. Since the 1970s, TMD has been proposed to have a multifactorial etiology in which various contributing factors are responsible for the pain and dysfunction [4,5]. These factors include structural conditions, psychological morbidity, and behavioral problems such as parafunctional habits [6,7].

Sugisaki et al. [8] reported that the prevalence of TMD-related symptoms (TRS) was higher in working population (approximately 17–18%) than in the general population (5–12%). They attributed this discrepancy to psychological irritation resulting from duties in the workplace, changes in the work environment, interpersonal relations, and an achievement-oriented climate in which an individual’s employment may be terminated if his or her job performance is perceived as weak or substandard. They also showed the necessity of investigating the associations between TRS and the work environment, business hours, amount of sleep, and other related factors. Although the *Ministry of Health, Labour and Welfare of Japan* reported in 2003 [9] that the annual Japanese business hours had decreased slightly over the previous 5-year period, this figure remains longer than that of the USA, the UK, Germany, or France [10]. *The Japan Institute for Labour Policy and Training* reported that approximately 60% of business establishments have at least 1 employee with a mental health problem and that the number of such employees has increased 30% between 2008 and 2010 [11]. Many studies suggest that psychosocial factors, including depression, stress, and anxiety, are involved in the predisposition to and initiation and perpetuation of TMD as well as in the response of TMD patients to treatment [12-17]. Visual display terminal (VDT) work time, such as personal computer (PC) use, has increased in recent years, and such an extension of VDT work time may influence musculoskeletal pain and mental health [18-21].

Therefore, among other factors, work environment factors such as work time, PC usage time, and commuting time influence TRS. Thus, the present study seeks to (a) investigate the prevalence of TRS in the working population, (b) investigate the effect of the work environment on TRS, and (c) identify the factors that contribute to TRS development.

## Materials and methods

This was a questionnaire-based cross-sectional study that maintained the anonymity of the respondents. This study was conducted with the approval (No. 325) of the ethics committee of Tokyo Medical and Dental University.

### Subjects and data collection

During the study period (April–October 2008), 2,723 employees of a Japanese company who underwent office medical check-ups participated in the study. The company develops, manufactures, and sells electronic parts and included a main office and a factory in Tokyo along with other factories in nearby prefectures. The employees were informed of the purpose and contents of our questionnaire before the study began. The questionnaire was distributed to all employees along with the notification of a medical check-up and was filled out before and then collected during the check-up. Of these employees, 2,423 (89%) answered the questionnaire. The subjects were informed that completing the questionnaire was optional and that by completing it they were consenting to the anonymous use of their answers in the present study. Written informed consent to participate was not obtained because individual identification was not necessary. The subjects were considered to have consented to participate in this study by answering the questionnaire.

### Questionnaire

Table 1 shows the questionnaire used in this study. The participants’ gender, age, and responses to questions 1–12 were recorded.

**Table 1:** Questionnaire

	**Question item**	**Abbreviated form**
Q1*	If you open your mouth wide, can you fit 3 fingers held vertically in your mouth?	Limited mouth opening
Q2*	Do you experience pain in the face, jaw, temple, or in front of the ear when you open and close your mouth?	Mouth-opening pain
Q3*	Can you open your mouth without any deviation?	Mouth-opening deviation
Q4*	Do you experience pain in the face, jaw, temple, or in the front of the ear when you eat chewy foods such as beef jerky, dried cuttlefish, or octopus?	Chewing-induced pain
Q5	How many hours per day do you use a personal computer at work? (h)	PC use
Q6	How many hours do you drive a car while working? (h)	Driving
Q7	How many hours per day do you perform precise work (e.g., working with parts by hand) while working? (h)	Precise work
Q8	How many hours does it take for you to commute to work? (h)	Commuting
Q9	How many hours per day do you spend at home before going to bed? (h)	Before sleep
Q10	How many hours do you sleep per day? (h)	Sleep
Q11	How many hours per day do you attend business meetings? (h)	Meetings
Q12	How many hours per day do you perform physical labor at work? (h)	Physical labor

*The subjects were asked to answer question items 1–4 on a 5-point numeric rating scale where 1 = strongly agree, 2 = weakly agree, 3 = neither agree nor disagree, 4 = weakly disagree, and 5 = strongly disagree.

Sugisaki et al. [22] developed questions 1–4, which were used to screen the subjects for TRS. The subjects were asked to rate these 4 screening question items using a 5-point numeric rating scale. Sugisaki et al. extracted these 4 items from a 20-item questionnaire previously administered to 2,360 dental patients. The sensitivity, specificity, and false-positive rate of TRS screening by the 4 questions used in this study were 0.746, 0.811, and 0.189, respectively. One item related to joint noise was not included in the screening questionnaire because its inclusion decreased the validity of the screening by item response theory analysis (Mokken analysis).

The other 8 questions were used to investigate the work environment. Questions 5–12 solicited information about the mean time spent each day on the following: using a PC at work, driving a car while working, performing precise work (i.e., working with small parts by hand), performing physical labor at work, attending business meetings, commuting, time spent at home before going to bed, and sleeping.

### Statistical analysis

The questionnaires collected from 454 respondents were excluded from the statistical analyses because of missing data. The data from the remaining 1,969 participants (81.3%) were analyzed.

The subjects’ ages and the mean values of the work environment parameters were compared between the low- and high-TRS groups using a *t*-test and the proportions of men and women using the Chi-square test.

The factors that may have influenced TRS were estimated by logistic regression analyses using odds ratios (OR) and 95% confidence intervals (CI) as measures of association. The covariates were entered into the logistic regression analysis by a stepwise forward technique with *P* < 0.05 considered statistically significant. An OR ≥2 or ≤0.5 was considered a clinically meaningful predictor. All statistical analyses were performed using the Statistical Package for Social Sciences (SPSS) (version 12.0, Tokyo).

## Results

### Comparison of the work environment between the low- and high-TRS groups

The median total score of the responses to questions 1–4 was 5 (25% = 4, 75% = 7). Two groups were defined based on the scores: participants scoring ≤7 were assigned to the low-TRS group and those scoring ≥8 to the high-TRS group.

Table 2 shows the subjects’ background characteristics and work environment parameters with respect to their TRS scores. Of the 1,969 employees, 445 (22.6%) were considered to have high TRS scores. Women were significantly overrepresented in the high-TRS group (19.6%) relative to the low-TRS group (12.2%), and the mean age of the high-TRS group (39.7 ± 8.7 years) was significantly younger than that of the low-TRS group (41.3 ± 9.8 years).

**Table 2:** Subjects’ background and work environment parameters by TRS score group

**Subjects’ background and work environment parameters**	**Low-TRS group (score ≤7)**	**High-TRS group (score ≥8)**	**Total**	***P*-value**
Subjects (%)	1524(77.4)	445 (22.6)	1969 (100)	-
Gender (% women)	186 (12.2)	87 (19.6)	273 (13.9)	< 0.001 ^a^
Age: mean (SD)	41.3 (9.8)	39.7 (8.7)	41.0 (9.6)	0.001 ^b^
PC use (h): mean (SD)	5.16 (2.84)	5.51 (2.89)	5.24 (2.85)	0.022 ^b^
Driving (h): mean (SD)	0.57 (0.70)	0.57 (0.83)	0.57 (0.73)	0.932 ^b^
Precise work (h): mean (SD)	0.97 (1.65)	1.08 (1.62)	1.00 (1.65)	0.210 ^b^
Commuting (h): mean (SD)	1.32 (0.95)	1.25 (0.94)	1.31 (0.95)	0.129 ^b^
Before sleep (h): mean (SD)	3.49 (1.30)	3.59 (1.32)	3.51 (1.31)	0.150 ^b^
Sleep (h): mean(SD)	5.95 (1.05)	5.91 (1.00)	5.94 (1.03)	0.471 ^b^
Meetings (h): mean (SD)	0.88 (0.99)	0.78 (0.92)	0.86 (0.98)	0.046 ^b^
Physical labor (h): mean (SD)	0.20 (0.65)	0.19 (0.51)	0.20 (0.62)	0.715 ^b^

TRS, temporomandibular disorder-related symptoms; PC, personal computer; SD, standard deviation; a, Chi-square test; b, *t*-test.

The PC use time (mean ± standard deviation [SD]) was significantly longer in the high-TRS group (5.51 ± 2.89 h) than in the low-TRS group (5.16 ± 2.84 h), and the meeting time (mean ± SD) was significantly shorter in the high-TRS group (0.78 ± 0.92 h) than in the low-TRS group (0.88 ± 0.99 h). No other work-related variables differed significantly between the 2 groups.

### Work environment factors that influence TRS

Table 3 shows the correlation coefficients for the work environment parameters. As none of the correlation coefficients were high, we used the answers to all questions as independent variables in the logistic regression analyses.

**Table 3:** **Correlation coefficients (*P*-values) for work environment parameters**

	**Driving**	**Precise work**	**Commuting**	**Before sleep**	**Sleep**	**Meeting**	**Physical labor**
PC use	−0.229	−0.149	0.163	−0.115	−0.146	0.198	−0.251
	(<0.001)	(<0.001)	(<0.001)	(<0.001)	(<0.001)	(<0.001)	(<0.001)
Driving	-	0.072	−0.023	0.071	0.061	−0.029	0.153
		(0.002)	(0.32)	(0.002)	(0.008)	(0.208)	(<0.001)
Precise work	-	-	−0.045	0.030	−0.031	−0.079	0.205
			(0.047)	(0.194)	(0.182)	(0.001)	(<0.001)
Commuting	-	-	-	−0.164	−0.137	0.049	−0.070
				(<0.001)	(<0.001)	(0.031)	(0.002)
Before sleep	-	-	-	-	0.089	−0.206	0.043
					(<0.001)	(<0.001)	(0.042)
Sleep	-	-	-	-	-	−0.041	0.005
						(0.073)	(0.836)
Meeting	-	-	-	-	-	-	−0.058
							(0.012)

Table 4 shows the results of the logistic regression analyses (low-TRS, 0; high-TRS, 1). Only statistically significant independent variables (*P* < 0.05) are shown. Gender (OR, 1.62; 95% CI, 1.21–2.27) and PC use time (2-h incremental OR, 2.23; 95% CI, 2.14–2.31) contributed significantly to the manifestation of high TRS scores. The ORs for PC use time are presented for 2-h increments, 1-h increments, and 0.1-h increments. Although a 0.1-h increment did significantly increase or decrease the OR, the OR was not clinically meaningful (≥2.0 or ≤0.5).

**Table 4:** Results of logistic regression analysis by a stepwise method

**Independent variable**	***P*-value**	**Odds ratio**	**95% confidence interval**
Gender: male	0.001	1	
: female		1.62	1.21–2.17
PC usage	0.005		
0.1 h increments		1.04	1.00–1.08
1.0 h increments		1.49	1.44–1.55
2.0 h increments		2.23	2.14–2.31

## Discussion

With regard to age and meeting time, there were significant differences in PC use time between the low-TRS group and the high-TRS group. However it is difficult to discern the significance of this finding because the mean differences were very small (age = 1.6 years, meeting time = 0.1 h).

The PC use time was longer for the high-TRS subjects than for the low-TRS subjects. Each additional 2 h of a subject’s mean PC use time increased the subject’s TRS morbidity 2.23-fold, e.g., subjects who used PCs 4 h per day had 2.23-fold higher rates of TRS than subjects who used PCs 2 h per day. This finding suggests that prolonged operation of a PC influences the manifestation of TRS.

A gender difference was observed in the present study in that women formed a significantly larger proportion of the high-TRS group (19.6%) than the low-TRS group (12.2%) and exhibited 1.62-fold greater TRS morbidity than men. Women have been reported to predominate among patients requiring treatment for TMD [23]. Moreover, a relationship between female sex and painful TMD symptoms has been widely reported in the literature [24-26]. Horowitz and Sarkin ascribed the high prevalence of TMD in women to their higher-frequency of PC use [27]. In the present study, the mean (SD) PC use time was significantly higher for women (6.0 [2.9] h) than for men (5.1 [2.8] h; *t*-test, *P* < 0.001). As the incidence of TMD in the general population has been reported to be 5–12% [2,3], the correlation between TRS and work environment seems clear, and many of the subjects were PC users.

Perri et al. reported an association between the number of years of PC use and symptoms of TMD [28]. Horowitz and Sarkin have estimated that ≥75% of the approximately 30 million Americans who suffer from TMD are women and ascribed this finding to women’s high frequency of PC use both at home and in the workplace [27]. Horowitz and Sarkin believe that VDT work such as PC use might be linked to 3 additional sympathetic nervous system irritants: (1) electrostatic ambient-air negative-ion depletion, (2) electromagnetic radiation, and (3) eyestrain and postural stress associated with poor work habits and improper workstation design.

To our knowledge, the association between the duration of daily use of PC and TRS (or TMD) has not been investigated, although the use of apparatuses equipped for VDT work has been monitored. The widespread availability of PCs has led to VDT use in many situations, including the workplace, thereby increasing the number of individuals exposed to these systems. Nakazawa et al. [29] reported that the likelihood of developing physical symptoms such as headaches, neck pain, back pain, and eyestrain increased when daily exposure to VDTs exceeded 3 h and that mental and sleep disorders could be prevented by restricting the use of VDTs to ≤5 h/day [29]. In addition, many researchers have reported that prolonged and uninterrupted daily VDT use causes eyestrain and musculoskeletal pain, both of which are associated with deterioration in mental health [18-21].

In patients with facial pain or with TMD symptoms, emotional stress might play a role in the etiology of oral habits such as tooth clenching, and the masseter muscle may be active during a stressful event [30,31]. In 2006, Sato et al. named daytime light tooth clenching activity the “tooth-contacting habit” (TCH) [32]. The TCH is defined as a habitual behavior in which the upper and lower teeth are continuously brought together with minimal force in a non-functional situation (i.e., contact rather than clenching). Cheng et al. used a radio wave-activated wrist vibrator to show that patients with myogenic pain had an incidence of non-functional tooth contact during the day nearly 4 times that of healthy controls [33]. Using a questionnaire, Michelotti et al. also reported that the habit of keeping the teeth in contact was a significant risk factor for myofascial pain [34]. According to the results of our previous study on the same workforce, TRS was associated with increases in both anxiety and habitual behaviors such as sleep bruxism (based on associated morning symptoms) and daytime light teeth clenching [16]. Michelotti et al. reported that the activity of the masticatory muscles was higher in the intercuspal position than at rest [35]. Continuous non-functional tooth contact is therefore thought to overload the temporomandibular joint and masticatory muscles. In fact, the association between clenching and muscle pain has been shown to be attributable to either muscle fiber damage or a reduction in the blood supply to the fibers due to significantly lower perfusion of the masseter [36-39]. The TCH is also thought to affect the onset, persistence, and aggravation of TMD. Habitual behaviors such as the TCH are considered nervous habits, that is, behaviors that are aggravated by tension or performed to reduce tension [40,41]. VDT work such as PC use or detailed work may increase habitual behavior such as the TCH. This result suggests that management of PC usage time is important for both employers and employees to reduce a risk factor for TRS.

PC use probably influences TRS indirectly. In other words, it is possible that TRS is actually related to increased incidences of habitual behaviors such as the TCH that are more often performed during PC use. Future work is required to examine the associations between PC use, detailed work, and the TCH. As this study was carried out at a single company, it may not apply to other working populations.

## Conclusions

The present questionnaire-based study showed that gender and PC use time was associated with TRS in the working population. Thus, evaluation of ergonomics is suggested for TMD patients.

## Abbreviations

TRS: Temporomandibular disorder-related symptoms; TMD: Temporomandibular disorders; VDT: Visual display terminal; CI: Confidence interval; TCH: Tooth contacting habit; PC: Personal computer; OR: Odds ratio.

## Competing interests

The authors declare that they have no competing interests.

## Authors’ contributions

AN and KT carried out the study. KK performed the statistical analysis. MS advised about statistics. All authors read and approved the final manuscript.
